# Avoiding the cost of complacency: barriers and solutions to ending the HIV epidemic in Europe

**DOI:** 10.3389/fpubh.2026.1814445

**Published:** 2026-09-16

**Authors:** Priscila Radu, Helen Hayes, Ellie Tunnicliffe, David Mott, Grace Hampson

**Affiliations:** Office of Health Economics (OHE), London, United Kingdom

**Keywords:** HIV - Human Immunodeficiency Virus, burden of disease, antiretroviral therapy, UNAIDS targets, long-acting therapies

## Abstract

**Introduction:**

Despite significant advances in antiretroviral therapy and prevention strategies, Europe remains off track to meet the UNAIDS 2030 targets of ending HIV as a public health threat. This study explores the implications of failing to meet these targets by quantifying the associated costs and identifying pathways to get back on track.

**Methods:**

We adopted a mixed-methods approach across eight European countries. Targeted literature reviews estimated annual and lifetime HIV costs, identified barriers and solutions, and assessed evidence on long-acting injectables (LAIs). Epidemiological data from UNAIDS and ECDC informed projections of HIV prevalence and diagnoses to 2030 under three scenarios. Semi-structured stakeholder consultations validated and expanded the evidence base, and an expert roundtable prioritized solutions and evaluated LAIs' potential impact.

**Results:**

If current trends persist, over 98,000 new HIV diagnoses are projected between 2025–2030, adding more than €4 billion in healthcare costs and nearly €36 billion in lifetime costs. Prevalence-related costs across five countries could reach €66.3 billion by 2030. Barriers occur at multiple levels, including individual (low risk perception, treatment adherence), societal (stigma, limited community engagement), healthcare (restricted service accessibility), and policy (insufficient funding and fragmented surveillance). LAIs emerged as a promising strategy to overcome adherence challenges and reduce stigma.

**Conclusion:**

Without intensified action, Europe faces escalating costs and rising diagnoses. Tackling structural barriers and diversifying treatment options is essential to altering current trajectories. LAIs offer a potentially transformative addition to the HIV care landscape, but achieving their impact requires equitable access, sufficient healthcare capacity, and sustained political commitment.

## 1 Introduction

Human Immunodeficiency Virus (HIV) remains a major global health challenge over four decades after its discovery. UNAIDS surveillance data indicate that more than 91 million people have acquired HIV to date, with an estimated 44 million

deaths and approximately 40 million people currently living with HIV (1). The virus undermines the immune system, leaving individuals vulnerable to opportunistic infections and malignancies. Without treatment, HIV can progress to Acquired Immunodeficiency Syndrome (AIDS) (2). Advances in antiretroviral therapy (ART) mean that a person living with HIV, who adheres to treatment and maintains viral suppression can live a full and healthy life. Furthermore, an undetectable viral load means that the individual is not at risk of onward transmission, also known as U = U (Undetectable = Untransmittable) (3).

In response to this evidence, the 2014 Joint United Nations Programme on HIV/AIDS (UNAIDS) launched a global framework to accelerate progress toward ending the HIV epidemic by 2030, setting ambitious targets to drive collective action (4). Central to this approach is the 95-95-95 target, seeking to ensure that 95% of people living with HIV know their status, 95% of those diagnosed receive treatment, and 95% of those on treatment achieve viral suppression (5). The strategy also sets additional targets, including reducing stigma and discrimination (6). However, a growing body of monitoring and evaluation literature has documented persistent shortfalls against these targets: by 2024, fewer than 90% of people living with HIV were on treatment and achieving viral suppression (1).

These clinical and public health gaps are compounded by a significant economic burden. Estimates suggest that the median lifetime cost of managing HIV in high income countries is $377,820 (7).

A considerable body of research has examined the barriers underlying these persistent gaps and proposed multi-level strategies to address them. Key initiatives include expanding access to testing, prevention, and diversified treatment in community-based, stigma-free settings; engaging communities with tailored education and support; combating stigma through inclusive public awareness campaigns; and strengthening data collection for vulnerable populations. These measures help overcome individual, societal, and systemic challenges impeding progress in the global HIV response (8–14).

While many strategies for strengthening the HIV response are well recognized, translating them into consistent progress across Europe has proven difficult, and the economic and human costs of this stagnation are not fully quantified at a European level. Long-acting injectable formulations represent a relatively new and underexplored approach to strengthening this response, with potential to reduce the daily burden of adherence and mitigate fear of disclosure and stigma (15). However, existing evidence has focused largely on clinical efficacy and acceptability, with limited exploration of their role in a European policy context (16, 17).

This study aims to understand the implications of failing to meet the UNAIDS 2030 targets by quantifying the associated costs and identifying pathways to get back on track. Specifically, this study addresses these gaps by estimating the economic burden of the epidemic's current trajectory, identifying and prioritizing key barriers to progress, and examining solutions including the potential role of using long-acting injectables (LAIs).

## 2 Methods

A mixed-methods approach was adopted to examine the current landscape of HIV prevention, treatment and policy. The geography of interest was Europe, with focus on eight countries (Figure 1): five key regional players (France, Germany, Italy, Spain and the UK), alongside three with relatively high HIV incidence, ranking 7th (Belgium), 3rd (Ireland), and 14th (Poland) out of 30 European countries based on HIV incidence rate (18).

**Figure 1:**
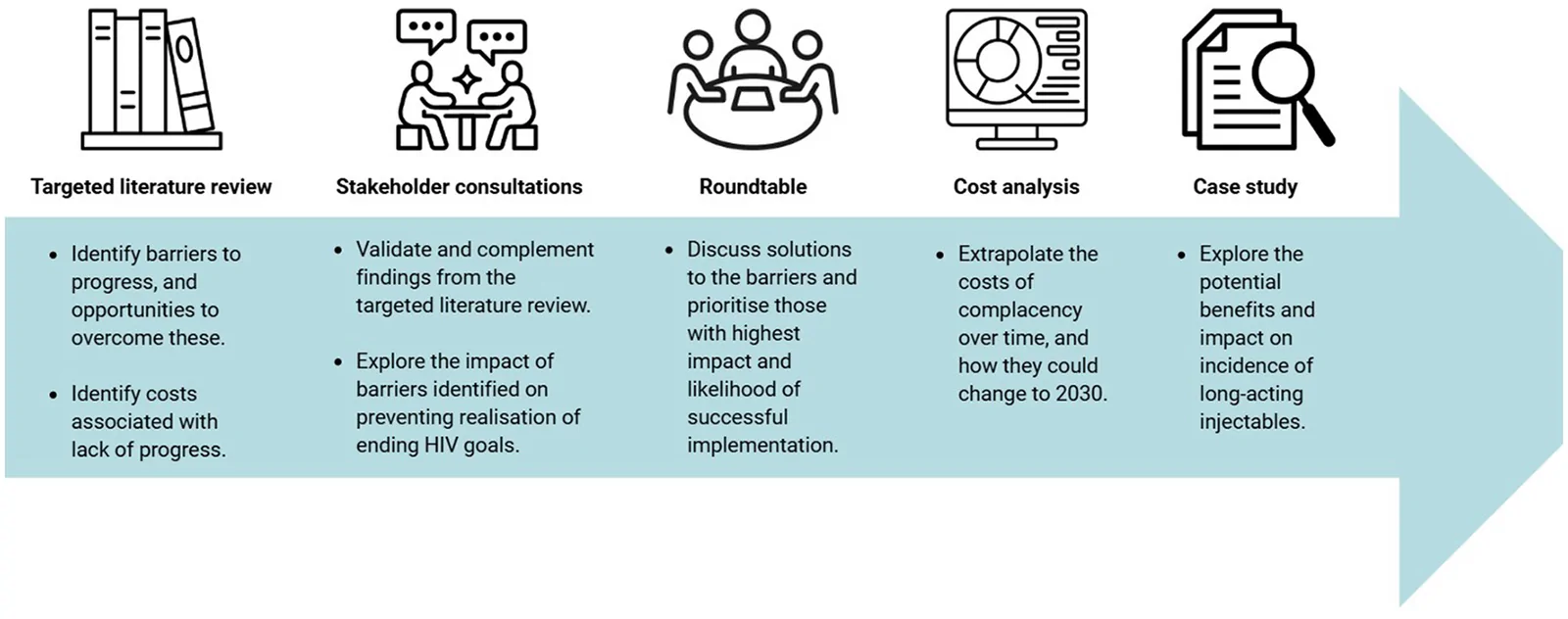
A visual representation of the methods

### 2.1 Cost of the status quo

We conducted a targeted literature review (TLR) to estimate annual HIV costs per person over the past decade. All costs were inflated to 2023 values using the Consumer Price Index (CPI) and converted to EUR using annual average exchange rates, obtained from the European Central Bank Data Portal (19). Detailed cost data by country are provided in Supplementary Table 1.

We contextualized these costs by examining HIV prevalence using data from UNAIDS (20) and diagnoses using data from ECDC (18) between 2014–23. Prevalence data captures the estimated total number of people living with HIV (diagnosed and undiagnosed) and associated treatment demand. Diagnosis data indicates new, diagnosed, reported infections, reflecting transmission dynamics and challenges for healthcare systems. Diagnosis data covers all of the countries in scope; prevalence data is only available for France, Spain, Italy, Germany, Belgium, the UK and Ireland.

We projected country-specific trends to 2030 by extending linear trends observed over different time periods based on data stability. For prevalence, we extrapolated the 2014–23 trend only, given the stable historical pattern. For diagnoses, we produced three scenarios: (1) extending the 2014–23 trend to reflect longer term historical patterns; (2) extending the 2019–23 trend to capture recent acceleration in diagnosis rates; and (3) extending the 2020–23 trend to present a continuation of post-pandemic trends.

We estimated the cost of the status quo, defined as the health expenditure burden of new infections if current efforts are not intensified. For prevalence, we multiply the projected number of people living with HIV between 2025–30 by the most recent per-person annual healthcare costs identified in the TLR.

We calculated two *cost of status quo* measures for new HIV diagnoses projected between 2025 and 2030. First, we estimated cumulative healthcare costs during the projection period by applying annual per-patient costs to each new diagnosis from their diagnosis year through 2030. Second, we estimated lifetime cost burden by multiplying total projected new diagnoses across 2025–30 by the median lifetime cost of HIV management in high-income countries (7).

### 2.2 Barriers and solutions

We conducted TLRs to examine barriers and solutions to HIV progress. Searches were run in PubMed and Google Scholar using combinations of HIV-related terms (e.g., “HIV,” “PrEP,” “ART”) with barrier- and solution- related terms (e.g., “stigma,” “access,” “adherence”), restricted to English-language sources published from 2019 onwards and focused on the eight study countries or Europe-wide evidence. Peer-reviewed articles were included where they reported empirical findings, alongside evidence, or policy analysis relevant to HIV prevention, diagnosis, treatment, or care. Opinion pieces and sources without primary or synthesized evidence were excluded. Identified barriers and solutions were extracted into a structured matrix and categorized into four pre-specified domains: individual, societal, healthcare, and policy and research, adapted from established frameworks for health system analysis.

We then conducted two rounds of structured stakeholder consultation to validate and refine the barriers and solutions identified through our literature review. The first round involved clinicians, policymakers and payers from Italy, Spain, Portugal and the UK, who assessed the completeness and relevance of the barriers identified. The second round involved seven international patient advocates from the Netherlands, Portugal, Spain and the UK, whose frontline perspective on the system-level barriers facing people living with HIV was used to further refine this assessment. Finally, we convened a roundtable with experts to prioritize the barriers and solutions according to their impact and feasibility. Insights from this stakeholder engagement informed the synthesis presented in the subsequent sections.

### 2.3 Long-acting injectables case study

We conducted TLRs to explore the impact of LAIs on incidence and their potential to address some of the shortcomings of frequent or daily prevention and treatment interventions to improve adherence. This was supplemented with insights from the aforementioned interviews and roundtable.

## 3 Results

### 3.1 The cost of the status quo

#### 3.1.1 Projected prevalence trends

Increasing prevalence, driven by both new infections and improved survival among people already living with HIV, is projected to increase the cost of the status quo across the study countries between 2025 and 2030 (Figure 2). Detailed prevalence trend data are provided in Supplementary Table 2.

**Figure 2:**
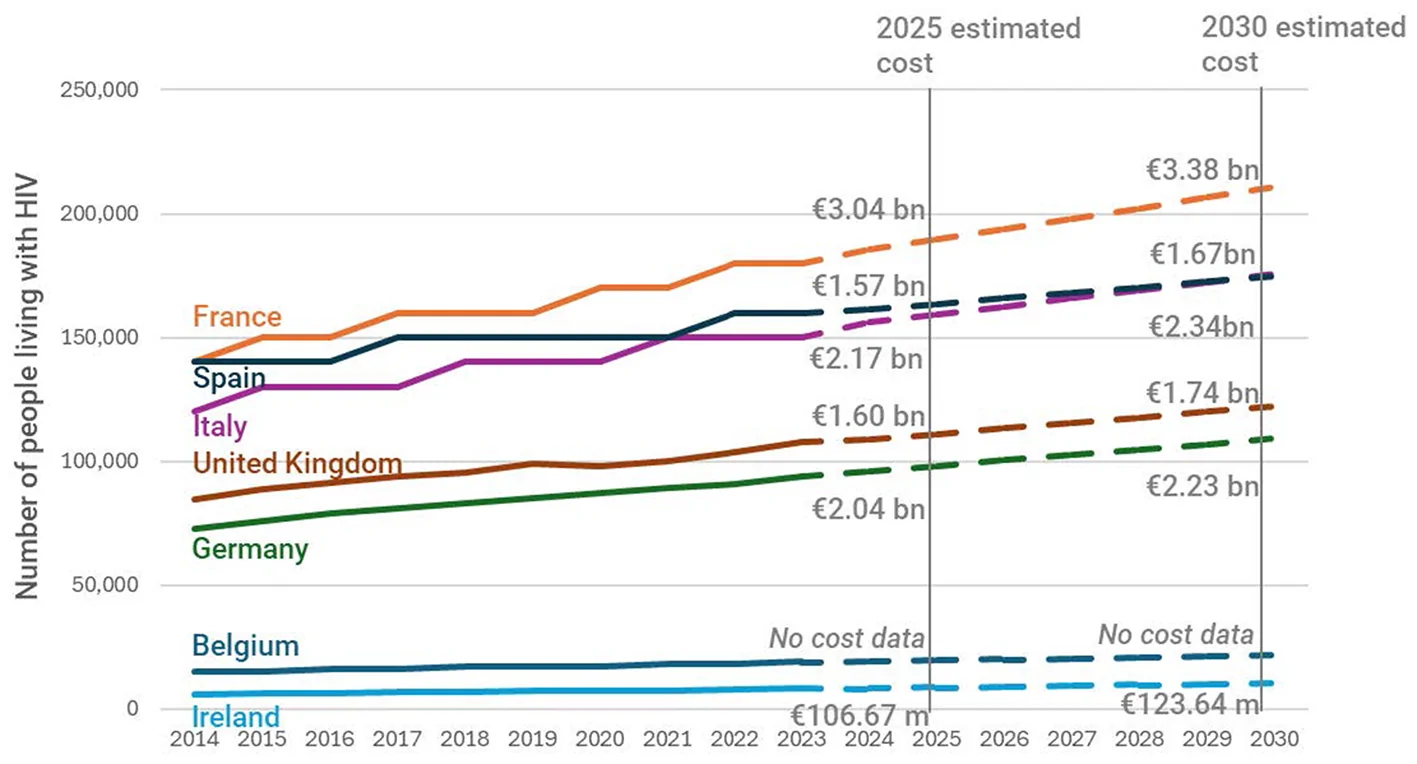
Projected population and associated cost of the status quo of people living with HIV (estimated diagnosed and undiagnosed). The solid line represents reported estimates (20). The dashed line illustrates a projected trend, calculated by extending the linear trend observed between 2014 and 2023 forwards to 2030. The estimated cost of care is calculated by the annual number of people living with HIV multiplied by the annual estimated cost per person living with HIV (Table 2). UNAIDS data does not include estimates for Poland. Annual cost estimates are unavailable for Belgium.

#### 3.1.2 Projected prevalence costs

Between 2025 and 2030, we estimate the cost of the status quo to increase by a range of 6.9–15.8% in the countries included in the study (Table 1). The total estimated cost of care between 2025 and 2030, calculated as the sum of the cost of the status quo across these 5 years, in France, Germany, Ireland, Italy, the UK and Spain is estimated at €66.3bn.

**Table 1:** Estimated costs of care for 2025, 2030 and combined cost across the 5 years.

Costs	Belgium	France	Germany	Ireland	Italy	Poland	Spain	UK
Estimated cost of care 2025	–	3.04bn	2.04bn	106.75m	2.17bn	–	1.57bn	1.60bn
Estimated cost of care 2030	–	3.38bn	2.27bn	123.64m	2.39bn	–	1.67bn	1.74bn
% increase	–	11.19%	11.24%	15.83%	10.27%	–	6.85%	8.58%
Estimated cost of status quo (2025–30)	–	19.25bn	12.96bn	691.17m	13.66bn	–	9.72bn	10.00bn

UNAIDS data does not include estimates for Poland. Annual cost estimates are unavailable for Belgium.

#### 3.1.3 Projected diagnosis trends

Country-level projections of diagnosis numbers, based on historical data, show a concerning recent uptick in newly diagnosed HIV cases (Figure 3). A sharp decline in diagnoses in 2020 reflects the potential impact of COVID-19 disruptions, followed by a notable increase in 2022–23 in several countries. This may partially reflect delayed or missed diagnoses during the pandemic. However, these missed cases may also have contributed to higher transmission rates among vulnerable populations unaware of their status. Other factors, such as changes in testing coverage, access to timely diagnosis and migration patterns, may have also influenced the recent uptick (18).

**Figure 3:**
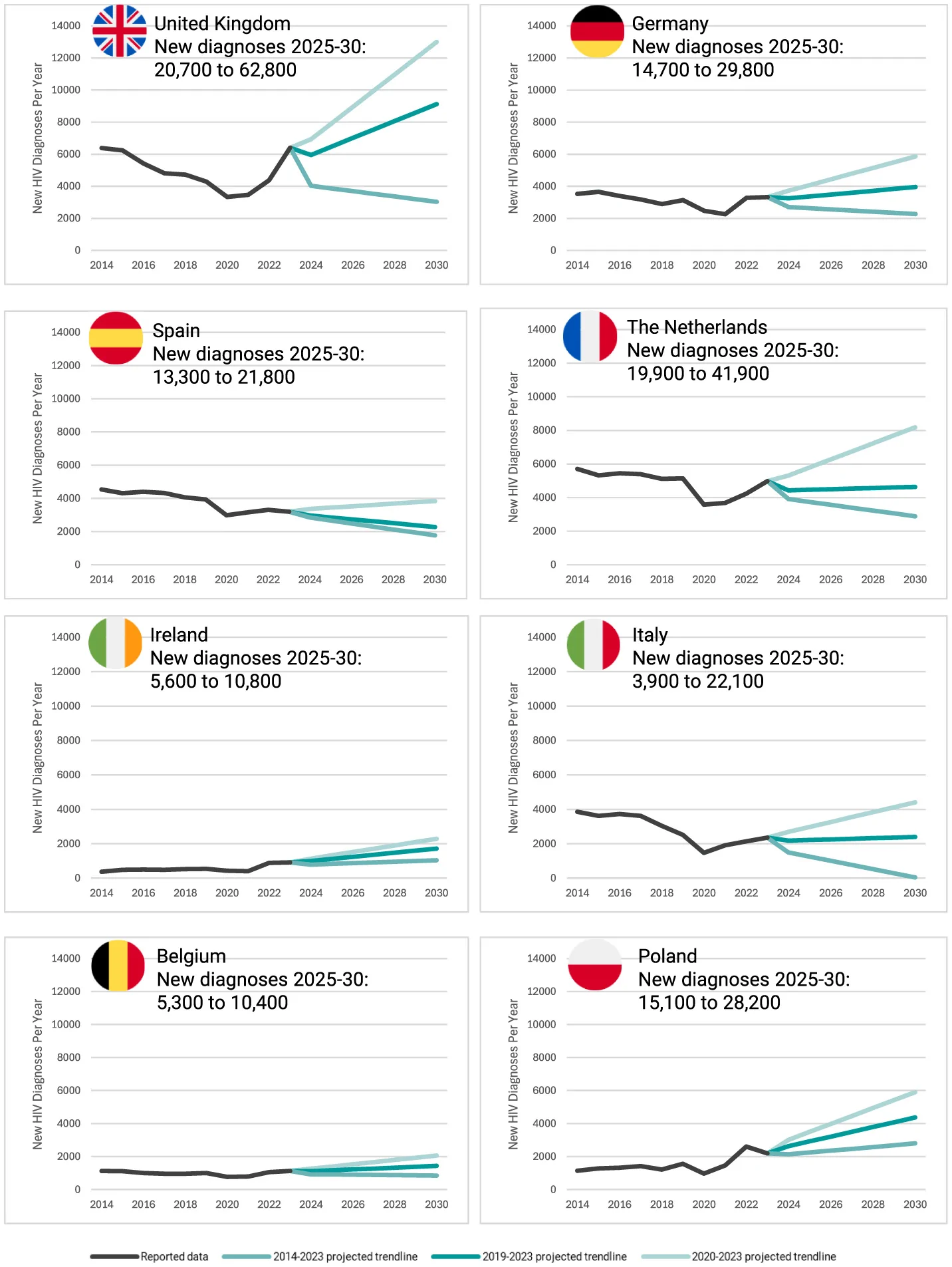
Projected number of new HIV diagnoses per year. Dark gray lines present actual reported data. The three colored projections correspond to projected trends based on an extrapolated linear trend of 2014–2023, 2019–2023 and 2020–2023 (18).

Based on the 2019–2023 projection, the UK, Poland and Ireland show the steepest future trajectory in diagnoses. Assuming trends follow a more conservative trajectory based on 2014–23 data, there is still a projected increase in Ireland, Poland and Belgium. No country is on track to reach zero new diagnoses by 2030 based on any trajectory.

#### 3.1.4 Projected diagnosis costs

Applying the most conservative projection (based on 2014–23 trends), new HIV diagnoses between 2025 and 2030 are estimated to generate substantial additional healthcare and lifetime costs across the study countries (Table 2).

**Table 2:** Estimated annual healthcare cost and projected lifetime costs of new infections between 2025 and 2030.

Costs	Belgium	France	Germany	Ireland	Italy	Poland	Spain	UK	Total		
									2014–23 trend	2019–23 trend	2020–23 trend
Estimated annual healthcare system cost pp (EUR)	–	16,030	20,833	12,095	13,597	2,637	9,577	13,802	n/a	n/a	n/a
Total new diagnoses (2025–2030)	5,270	19,868	14,712	5,587	3,870	15,123	13,295	20,679	98,405	163,399	227,840
Additional costs (incurred in 2025–2030)	–	1.16bn	1.10bn	228m	242m	134m	475m	1.04bn	4.38bn	6.92bn	9.41bn
Additional costs (lifetime for new diagnoses within 2025–2030 period)	1.91bn	7.19bn	5.32bn	2.02bn	1.40bn	5.47bn	4.81bn	7.48bn	35.59bn	59.10bn	82.41bn

Estimated healthcare system costs per person (pp) are not directly comparable as they are retrieved from various sources. However, of all costs considered, healthcare system cost components offered the highest level of comparability. Total figures have not been presented for the annual healthcare system cost per person, as these are component costs used in the calculation of the cost of the status quo. All costs have been adjusted to 2023 EUR. Annual cost estimates are unavailable for Belgium.

Median lifetime cost of managing HIV in high income countries is used for all countries (7) (377,820 2021 USD = 361,712.05 in 2023 EUR).

The total additional cost between 2025 and 2030 is estimated to amount to more than €4bn across all countries, and the additional lifetime costs of new infections occurring between 2025 and 2030 could reach nearly €36bn across all countries.

### 3.2 Barriers and solutions

We categorized the barriers by the levels at which they take place: individual, societal, healthcare & policy, and research. This framework makes it easier to identify where interventions are most needed and to tailor solutions to each level in a systematic way.

#### 3.2.1 Individual barriers

Individual barriers are personal factors that limit someone from effectively engaging in behaviors or accessing services designed to reduce their risk of HIV or for HIV treatment. Individuals' personal knowledge of HIV emerged as a key concern, particularly regarding low perceived risk and limited awareness of available services (11, 21). These challenges are further compounded by language barriers and mistrust in healthcare systems, which are disproportionately experienced by underserved populations (10–12).

Comprehensive school-based sexual education programmes is key to foster a culture of safe practice and awareness in a step toward addressing these barriers (10, 22). Strengthening collaboration with communities, particularly those comprising at-risk groups, and disseminating educational material can further help to address misconceptions surrounding transmission, management and treatment of HIV (8).

#### 3.2.2 Societal barriers

Societal barriers encompass challenges rooted in prevailing norms and expectations. Among these, stigma remains particularly pervasive and detrimental due to its far-reaching negative effects across the HIV care continuum. It discourages testing, delays treatment initiation, reinforces discrimination, and perpetuates misinformation—ultimately sustaining transmission and worsening health outcomes (8, 10, 14, 23–26).

Addressing these societal barriers requires targeted interventions that directly confront stigma. Evidence highlights several impactful and feasible approaches, including decriminalization of HIV transmission and behaviors unjustly associated with HIV such as drug use and sex work (11, 27), the implementation of opt-out testing in high-prevalence areas (13), and mandatory education for healthcare providers on HIV transmission, infection control, and stigma reduction (13). Training clinicians to routinely offer HIV tests based on clinical presentation, irrespective of other factors, such as gender, sexuality or race may further contribute to normalizing testing practices and reducing stigma within healthcare settings (8).

#### 3.2.3 Healthcare barriers

Healthcare barriers arise from systemic shortcomings within current healthcare structures, including in clinical guidelines, staff training and access to medication. These barriers primarily concern the availability of, and access to, diagnostic, treatment, and preventive services, both within and beyond traditional clinical settings (10, 28). Limited accessibility outside medicalised environments can disproportionately affect populations with reduced engagement in healthcare systems, such as marginalized or socioeconomically disadvantaged groups (29–33).

Expanding service delivery beyond conventional healthcare settings offers a promising avenue to overcome these barriers. For instance providing pre-exposure prophylaxis (PrEP) through community-based organizations could substantially improve uptake among at-risk populations (10, 22). Similarly, broadening the range and accessibility of HIV testing modalities, such as enhancing awareness and distribution of home testing kits, could strengthen early diagnosis and linkage to care (10, 34).

#### 3.2.4 Policy and research barriers

Policy and research barriers stem from limited resources and structural weaknesses within political and academic systems. Insufficient government commitment and inconsistencies in surveillance and data reporting within and across countries remain major obstacles to advancing the HIV agenda (10, 35).

Sustained political commitment is critical to achieving HIV elimination goals in Europe, as underscored by calls for a renewed EU Action Plan (36), consensus at the European Parliament over EU actions needed (37), ongoing WHO regional efforts (38), and G7 leaders' reaffirmed commitment to ending HIV by 2030 (39). Strengthening financial support for HIV research and innovation, alongside establishing interoperable data-sharing systems to standardize and integrate surveillance across countries, will be essential to accelerating progress (10). Reducing HIV incidence remains central to achieving global targets for epidemic control. While a broad range of potential solutions exists, only a limited subset is supported by quantifiable data directly linked to HIV incidence.

Roundtable discussions highlighted better testing as key to progress, yet evidence of its direct impact on new infections is limited. Data on stigma are also incomplete, and its complexity makes measuring its effects difficult. These gaps hinder a full quantitative assessment of the proposed solutions in reducing HIV transmission. Nonetheless, diversifying treatment options emerged as a promising avenue, warranting further investigation.

### 3.3 The potential impact of long-acting injectables

Long-acting injectables (LAIs) are a type of formulation with durations of action and administration frequencies of up to 6 months. The prolonged duration of action means that the active substance is released slowly over time after injection, allowing for less frequent dosing compared to oral alternatives. In the context of HIV, LAIs are available for both prevention and treatment. Evidence on oral long-acting HIV formulations is still early-stage, but emerging clinical trial data, particularly for once-weekly regimens, show progress toward effective, patient-preferred alternatives to daily pills (40).

PrEP is an important and effective intervention for preventing new HIV infections, and, when taken consistently, oral daily PrEP has been shown to reduce HIV risk by up to 92% (41). However, despite its proven efficacy, limited uptake and suboptimal adherence continue to undermine its population-level impact. In 2024, 344,596 individuals received PrEP across 36 reporting countries within the WHO European Region–falling significantly short of the 500,000 target by 2025 (42). A recent meta-analysis found that 37.7% of individuals discontinued PrEP within 6 months of initiation, and among those who continued, over 30% used it with a frequency that was insufficient to provide protection (43).

ART is fundamental for people living with HIV, enabling viral suppression, preserving immune function, and preventing disease progression (44). By maintaining an undetectable viral load, ART also prevents onward transmission, forming the basis of the “treatment as prevention” strategy (3). Nevertheless, one in six people living with HIV, in the WHO European region, have still not achieved viral suppression (6). Most ART regimens require lifelong, daily oral administration, which can pose challenges similar to those observed with PrEP, including treatment fatigue, medication burden, and lifestyle-related adherence barriers (45). In the US, over 60% of people living with HIV demonstrate adherence rates below 90%, and more than 40% fall below 80% (46). Across Europe, 62.4% of respondents to a survey reported missing their medication more than once in the previous month, and 16.4% reported missing it more than five times (24). A large retrospective study across 31 countries reported that only 79% of adults achieved virologic suppression 1 year after initiating oral ART, declining to 65% at 3 years, highlighting the progressive erosion of treatment success in routine care (46).

Long-acting formulations offer a promising alternative that directly addresses many of these adherence barriers. By substantially reducing dosing frequency and eliminating the need for daily pill-taking, LAIs alleviate behavioral and psychological burdens associated with oral regimens (15). LAI-PrEP also offers important clinical advantages, serving as an option for individuals with contraindications to oral PrEP, such as renal impairment or reduced bone mineral density, thereby expanding access to prevention among populations previously excluded (47). Moreover, LAI PrEP minimizes the risk of drug–drug and drug–food interactions (48) and may help reduce stigma by decoupling HIV prevention from daily medication routines. For some people living with HIV, the discrete nature of LAI may be of importance due to the fear of disclosure of sexual orientation or HIV status associated with oral therapies, where the need to store pill bottles at home and take daily medication can inadvertently reveal status to partners, family members, or housemates. These features position LAIs as a promising strategy for reducing HIV incidence and advancing progress toward epidemic control.

#### 3.3.1 Impact on incidence

Evidence on the impact of LAIs remains limited due to their novelty. Most evidence comes from modeling studies, given the limited feasibility of retrospective analyses. Nonetheless, emerging research indicates promising effects of LAIs on reducing HIV incidence.

Although current evidence is largely based on modeling, projections from the United States suggest that LAI-PrEP could reduce annual HIV incidence among men who have sex with men (MSM) by 4–19% (49–51). Complementary analyses from Europe indicate that a rapid scale-up of LAI-PrEP among MSM in the Netherlands could avert 14–21% of new infections within 10 years of its introduction (45). A systematic review reported that LAI-PrEP reduced HIV risk by 79% compared with daily oral PrEP (16). Similarly, among HIV-uninfected women in sub-Saharan Africa, use of long-acting formulations was associated with an 88% lower risk of HIV acquisition relative to daily oral PrEP (52). These findings highlight the transformative potential of LAI-PrEP as a tool to substantially reduce HIV transmission and address persistent gaps in adherence and prevention coverage.

Unlike PrEP, which is indicated for individuals at risk of HIV, LAI-ART is currently only approved for people living with HIV who have already achieve sustained viral suppression. This regulatory limitation may underestimate its broader therapeutic potential, particularly among populations struggling with adherence, where viral load may not be adequately controlled (53). Despite this, evidence from multinational Phase 3 trials shows that monthly LAI-ART injections are non-inferior to daily oral regimens in maintaining viral suppression (54).

Modeling studies project that even restricting LAI-ART to those already supressed on oral therapy could reduce HIV incidence and increase population-level viral suppression. In a conservative scenario, assuming extended viral suppression without changes in adherence, HIV incidence decreased by up to 9%, and the proportion of diagnosed individuals achieving suppression increased from 69% to 73% (17). In a demonstration project involving participants with detectable viremia, LAI-ART not only sustained viral suppression among those already controlled on oral regimens, but also achieved virologic suppression in nearly all individuals who were unsuppressed at initiation (55). Expanding access to LAI-ART beyond the currently restricted population could help overcome adherence barriers, enhance viral suppression rates, and ultimately curb HIV transmission at the population level. Additional modeling is required to better estimate LAIs' potential public health benefit.

## 4 Discussion

### 4.1 Summary of findings

Europe stands at a critical juncture in its HIV response. Projections within this paper indicate that the region is not on track to meet the UNAIDS 2030 goal of zero new infections. Based on historical trends, over 98,000 people could be newly diagnosed with HIV between 2025 and 2030 across France, Germany, Ireland, Italy, Belgium, Spain, Poland, and the UK. Across all countries, the total additional cost of new diagnosed cases between 2025–30 is estimated to exceed €4bn, with lifetime costs reaching nearly €36bn. New infections and people living longer with HIV are projected to drive an increase in the total prevalence of people living with HIV (56). The projected cost of care associated with this, across just five of the countries studied, is estimated at €66.3bn. If current trajectories persist, ongoing transmission will further strain already overburdened healthcare systems.

Recent analyses similarly project only modest declines in HIV incidence and mortality, with rising prevalence and funding shortfalls threatening progress toward 2030 targets (57–59). Decisive action, anchored in sustained funding, stronger partnerships, and a revitalized focus on prevention, treatment and care, can safeguard Europe's health security and put the region back on course to end HIV as a public health threat by 2030.

### 4.2 Limitations

This study has several limitations. Prevalence results are based on modeled estimates, and using reported diagnostic numbers as a proxy for new infections likely underestimates the true incidence, due to surveillance gaps and undiagnosed or late diagnosed cases. Despite the concerning uptick in reported diagnoses, the modeled incidence estimates from UNAIDS (56) show a decreasing trend in infection rates since 2010. Rising diagnosis rates present immediate costs and capacity pressures. Linear extrapolation carries uncertainty and overlooks structural dynamics. We retained 2020–23 data across our projections, given the limited length of the underlying annual time series and the uncertainty around the long-term impact of the COVID-19 pandemic. Instead, we present three scenarios (2014–23, 2019–23, 2020–23) so readers can assess the sensitivity of projected trends to the inclusion of pandemic-affected years. Our cost projections should be read as indicative of the order of magnitude of the burden under current policy settings rather than precise forecasts. The central conclusion that current trajectories are inconsistent with the 2030 goals is robust to these uncertainties.

Second, the literature review was not conducted systematically, and the expert consultations were semi-structured, which may introduce selection or reporting biases. Third, variable cross-country data availability and unmodeled contextual factors, such as healthcare infrastructure, social determinants of health, and emerging epidemiological trends limit comparability and generalizability. As such, country-level cost figures should be treated as comparable in pattern rather than precise magnitude. We frame the findings as directional evidence to inform policy prioritization, with the call for change holding under plausible variations in the underlying assumptions.

### 4.3 Policy recommendations

We identify several strategies that could help realign Europe's HIV response, drawing on insights from literature and expert consultation. Expanding access to HIV services beyond traditional medical settings, and partnering with communities to co-design tailored education and support, would improve engagement and enable earlier diagnosis and prevention (38).

Combating stigma through co-designed, people-first public awareness campaigns targeting both the public and healthcare providers remains critical to timely diagnosis and prevention (8, 10, 13, 59). Equally important is strengthening data collection across Europe, particularly from vulnerable and hard-to-reach populations, to accurately capture the true burden of HIV (10, 12). These measures can enhance early diagnosis, improve prevention strategies, and ultimately deliver improved outcomes for those living with HIV.

Finally, diversifying therapy options is critical to meeting the diverse needs and preferences of individuals living with, or at risk of, HIV (24, 60). Long-acting therapeutic alternatives, which have demonstrated success and effectiveness in other therapeutic areas (61), offer greater convenience than daily oral regimens and may strengthen both individual and public health outcomes (15, 44, 62).

However, their adoption faces challenges, including logistical barriers from regular clinic visits, risk of missed doses, low awareness among users and providers, and structural inequities in access, particularly among marginalized populations (15, 55, 62–64). Current delays in oral PrEP initiation highlight the need to strengthen healthcare capacity, as LAI-PrEP requires additional resources for administration and monitoring. Moreover, equitable access remains a critical concern: structural inequities, stigma, systemic barriers, and declining multilateral funding threaten the widespread adoption of LAIs, particularly among marginalized populations (65). Addressing these multifaceted challenges is essential to ensure LAIs fulfill their promise in reducing HIV transmission globally.

### 4.4 Future prospects

Trends in HIV prevention and treatment have the potential to evolve rapidly. Since this analysis was conducted, the UK has become the first country in Europe to approve a long-acting injectable therapy for PrEP for specific populations within its National Health Service, demonstrating that policy innovation can accelerate access to new interventions (66). Emerging technologies, evolving policies, and shifts in social behaviors may further transform the landscape, enabling more effective prevention and care strategies than currently anticipated. While challenges remain, these developments allow us to be cautiously optimistic that concerted efforts and innovation can help put Europe back on track to achieving the 2030 targets for ending HIV as a public health threat.

## Data Availability

The original contributions presented in the study are included in the article/Supplementary material, further inquiries can be directed to the corresponding author.
