# Supplementary material for: Avoiding the cost of complacency: barriers and solutions to ending the HIV epidemic in Europe
Source: Front Public Health. 2026 Sep 16;14:1814445. doi: 10.3389/fpubh.2026.1814445 (PMC13624092; doi:10.3389/fpubh.2026.1814445)
Supplement: Supplementary file 1 [file Table_1.docx]

**Supplementary Table 1: Annual healthcare system cost data**

|  | Reference | Year | Original currency | Original Value | EUR | 2023 EUR |
| --- | --- | --- | --- | --- | --- | --- |
| Belgium (no data) | - | - | - | - | - | - |
| France | Prodel et al. 2021 | 2019 | EUR | 14,223.00 | 14,223.00 | 16,029.66 |
| Germany | Van de Vijver et al. 2019 | 2016 | EUR | 17,015.93 | 17,015.93 | 20,833.09 |
| Ireland | O murchu et al. 2021 | 2017 | EUR | 10,200.00 | 10,200.00 | 12,094.61 |
| Italy | Ferrario et al. 2020 | 2019 | EUR | 11,694.86 | 11,694.86 | 13,597.11 |
| Poland | Zah and Toumi, (2015) | 2013 | PLN | 7,629.98 | 1,817.74 | 2,636.98 |
| Spain | Lopez Segui et al 2023 | 2021 | EUR | 8,534.00 | 8,534.00 | 9,576.80 |
| United Kingdom | Ong et al. (2019) | 2017 | GBP | 9,743.67 | 11,114.41 | 13,802.25 |

|  | UNAIDs estimates | | | | | | | | | | Projected trend | | | | | | |
| --- | --- | --- | --- | --- | --- | --- | --- | --- | --- | --- | --- | --- | --- | --- | --- | --- | --- |
|  | 2014 | 2015 | 2016 | 2017 | 2018 | 2019 | 2020 | 2021 | 2022 | 2023 | 2024 | 2025 | 2026 | 2027 | 2028 | 2029 | 2030 |
| Belgium | 15,000 | 15,000 | 16,000 | 16,000 | 17,000 | 17,000 | 17,000 | 18,000 | 18,000 | 19,000 | 19,133 | 19,558 | 19,982 | 20,406 | 20,830 | 21,255 | 21,679 |
| France | 140,000 | 150,000 | 150,000 | 160,000 | 160,000 | 160,000 | 170,000 | 170,000 | 180,000 | 180,000 | 185,333 | 189,576 | 193,818 | 198,061 | 202,303 | 206,545 | 210,788 |
| Germany | 73,000 | 76,000 | 79,000 | 81,000 | 83,000 | 85,000 | 87,000 | 89,000 | 91,000 | 94,000 | 95,933 | 98,139 | 100,345 | 102,552 | 104,758 | 106,964 | 109,170 |
| Ireland | 5,800 | 6,100 | 6,300 | 6,600 | 6,900 | 7,100 | 7,300 | 7,500 | 7,900 | 8,600 | 8,547 | 8,826 | 9,105 | 9,385 | 9,664 | 9,944 | 10,223 |
| Italy | 120,000 | 130,000 | 130,000 | 130,000 | 140,000 | 140,000 | 140,000 | 150,000 | 150,000 | 150,000 | 156,000 | 159,273 | 162,545 | 165,818 | 169,091 | 172,364 | 175,636 |
| Poland | - | - | - | - | - | - | - | - | - | - | - | - | - | - | - | - | - |
| Spain | 140,000 | 140,000 | 140,000 | 150,000 | 150,000 | 150,000 | 150,000 | 150,000 | 160,000 | 160,000 | 161,333 | 163,576 | 165,818 | 168,061 | 170,303 | 172,545 | 174,788 |
| United Kingdom | - | - | 99,000 | 100,000 | 100,000 | 100,000 | 110,000 | 110,000 | 110,000 | 110,000 | 113,821 | 115,810 | 117,798 | 119,786 | 121,774 | 123,762 | 125,750 |

**Supplementary Table 2: Reported (2014-2023) and projected (2024-2030) prevalence estimates by country**

Notes: Reported estimates from UNAIDS are presented for the years 2014-2023. The years 2024-2030 extend the linear trend observed between 2014 and 2023 forwards to 2030 where data are available.
